# *Streptococcus mutans-*associated bacteria in dental plaque of severe early childhood caries

**DOI:** 10.1080/20002297.2022.2046309

**Published:** 2022-03-02

**Authors:** Yixin Zhang, Jiakun Fang, Jingyi Yang, Xiaolei Gao, Liying Dong, Xuan Zheng, Liangjie Sun, Bin Xia, Na Zhao, Zeyun Ma, Yixiang Wang

**Affiliations:** a Central Laboratory Peking University School and Hospital of Stomatology, Beijing, China; b Department of Oral and Maxillofacial Surgery, Peking University School and Hospital of Stomatology, Beijing, China; c National Engineering Laboratory for Digital and Material Technology of Stomatology, Peking University School and Hospital of Stomatology, Beijing, China; d Beijing Key Laboratory of Digital Stomatology, Peking University School and Hospital of Stomatology, Beijing, China; e Office of Operations Management, Peking University School and Hospital of Stomatology, Beijing, China; f Department of Pediatric Dentistry, Peking University School and Hospital of Stomatology, Beijing, China; g Department of Restorative Dentistry and Biomaterials Sciences, Harvard School of Dental Medicine, Boston, Massachusetts, USA; h Shanghai Stomatological Hospital, Fudan University, Shanghai, China; i Department of VIP Service, Peking University School and Hospital of Stomatology, Beijing, China

**Keywords:** Severe early childhood caries (SECC), 16S rRNA gene sequencing, *streptococcus mutans* abundance, dental plaque, bacteria

## Abstract

**Background:**

*Streptococcus mutans* (*S. mutans*) is a potential pathogenic bacteria of dental caries. However, the level of *S. mutans* is low in some children with severe early childhood caries (SECC)

**Aim:**

To evaluate the effect of *S. mutans* level on dental microbiome and cariogenesis.

**Methods:**

The oral microbiota was compared between caries-free group (CF) and SECC group.16S rRNA gene sequencing was used for *S. mutans* level bacterial community analysis. The candidate bacteria that were closely related with *S. mutans* abundance were identified and confirmed by absolute quantitative real-time PCR in clinical dental plaque samples from CF and SECC groups.

**Results:**

Through in-depth analysis of dental plaque microorganism, *Leptotrichia, Selenomonas* and *Prevotella_7* were found in the *S. mutans*-low group (*p* < 0.05) and *Porphyromonas, Selenomonas*_3 were found in the *S. mutans*-high group (*p* < 0.05). Through quantitative real-time PCR, *Leptotrichia, Selenomonas* and *Prevotella_7* were identified as the potential biomarkers of SECC when *S. mutans* was at a low level.

**Conclusion:**

*Leptotrichia, Selenomonas* and *Prevotella_7* are identified as potential biomarkers in SECC with a low abundance or without *S. mutans*. Our study may shed light on the understanding of caries occurrence in SECC with low abundance of *S. mutans*.

**Abbreviations:**

*S. mutans, Streptococcus mutans*; CF, caries-free; SECC, severe early childhood caries; ECC, early childhood caries; rRNA, ribosome RNA; qPCR, Quantitative real-time PCR; OTUs, operational taxonomic units; ANOVA, analysis of variance; LDA, Linear discriminant analysis; LEfSe, Linear discriminant analysis effect size; COG, Groups of proteins; NMDS, Non-MetricMulti-Dimensional Scaling; IL-1β, interleukin −1β; IL-6, interleukin-6; IL-8, interleukin-8; IL-10, interleukin-10.

## Introduction

Dental caries is a prevalent chronic disease, resulting from the demineralization of tooth tissues caused by acids produced from the bacterial fermentation of dietary carbohydrates. To date, dental caries remains a significant public health challenge. In 2017, the age-standardized prevalence of caries in deciduous teeth was 7.8%, while the number of prevalent cases reach to 532 million [1]. Early childhood caries (ECC) is defined as the presence of one or more noncavitated or cavitated lesions, caries-caused missing or filled surfaces, in any primary tooth of a child under six years old [2]. Early childhood caries (ECC) can cause serious oral problem as well as general health, including mouth pain and dental abscesses, impaired nutrition status, sleep disturbances [3,4]. Severe ECC (SECC) is an aggressive form of ECC. Based on the definition of SECC by the American Academy of Pediatric Dentistry (AAPD), children aged 3–5 years who have one or more cavitated lesions, caries-caused missing or filled smooth surface in primary teeth or decayed, missing or filled surfaces greater than or equal to four (age of 3), five (age of 4) or six (age of 5) are diagnosed as SECC patients. It occurs earlier in life, with more incidence and affects children growth, even physical and psychological health of the subjects during their whole lifespan [5].

The oral cavity harbors one of the most complex microbiomes in the body [6], and oral bacteria are one of the important causations of dental caries occurrence and progression. However, only about 50% of approximately 700 types of oral microorganisms have been cultivated. Caries is a multifactorial disease. It results from acids produced from the bacterial fermentation of dietary carbohydrates. Acid causes the demineralization of tooth tissues. A shifted balance of microbiota takes place in the oral environment during the caries process [7], in which bacterial members with the ability of acid-producing and acid-resisting could potentially initiate the occurrence of caries.

*Streptococcus mutans* (*S. mutans*) plays an important role in caries development for its strong ability of acid-producing and acid-tolerence and is regarded as the most common microorganism associated with ECC [8]. However, *S. mutans* was founded either at a low level or not present in some patients with caries [9–12] and was identified from the caries-free (CF) group, suggesting that other species, especially bacteria closely associated with *S. mutans*, may be responsible for caries development. Other bacteria, such as *Lactobacillus* spp [13,14], were considered to be correlated with dental caries apart from *S. mutans*. But most of the results were based on the analysis between healthy people and caries patients. Though some studies had confirmed that a significant proportion of the population has caries without detectable *S. mutans*. However, studies regarding *S. mutans* level-related bacteria by 16S rRNA gene sequencing were seldom [10,11]. Therefore, *S. mutans* level-related bacteria, which may contribute to the disease process in SECC, were investigated in this study to provide more evidence.

In our study, 16S rRNA gene sequencing was employed to compare the bacterial community composition of dental plaque from the children who suffered SECC and the children who were caries-free. According to the relationship between *S. mutans* and other bacteria in CF group and SECC group, we investigated the potential bacteria beyond *S. mutans* in SECC with low-level *S. mutans*. Moreover, we verified our candidate bacteria by absolute quantitative real-time PCR (qPCR) to determine the abundance of differential targeted bacteria in CF and SECC. As expected, *S. mutans* was the dominant species in many, but not all, subjects with caries. *Leptotrichia, Selenomonas* and *Prevotella* were observed as alternative pathogens which were significantly associated with caries. Our new findings provided a theoretical explanation for caries development and then may be helpful for targeted strategy toward to prevention and therapy of dental caries.

## Materials and methods

### Patient recruitment and sampling

The study is approved by the Ethics Committee of Peking University School of Stomatology (PKUSSIRB-201839140). Patients were consecutively recruited from the Department of Paediatric Dentistry at the School of Stomatology of Peking University with an informed consent form. Children who had systemic diseases, visually detectable enamel or dentin hypoplasia, a history of antibiotics or anti-inflammatory drug treatment within the preceding 2 weeks prior to the study, or a history of fluoride treatment within the preceding month prior to the study were excluded. 217 children aged from 2 to 6 years with full deciduous teeth were divided into two groups: Group CF, children who had no caries lesions or restorations (n = 132) and Group SECC, children diagnosed with severe early childhood caries (n = 95). Ten samples from each group were utilized for detecting dental plaque microorganism using 16S rRNA high-throughput sequencing. Others were used to validate the 16S rRNA high-throughput sequencing data. All parents of these children in the study gave informed consent to participate.

The teeth of all children participating in this study has been examined by using the ICDAS caries detection system. Participants were required to avoid eating, drinking and brushing their teeth for 2 h before collecting samples. The dental plaque samples were collected by a trained paediatric dentist between 9:00 and 10:00 a.m, from the labial smooth surfaces or the decayed cavity using probe. The dental plaque was transferred to 1.5 mL sterile centrifuge tube and stored at −80°C immediately until DNA extraction.

### DNA extraction and sequencing

DNA was extracted and purified using the TIANamp Bacteria DNA Kit (Tiangen Biotech, Beijing, China) according to the manufacturer’s instructions. The quality and concentration of total DNA were determined using a NanoDrop 8000 spectrophotometer (Thermo Fisher Scientific, Carlsbad, CA, USA). Integrity was checked by 1.8% agarose gel electrophoresis. The V3-V4 region of the bacterial 16S rRNA was PCR-amplified using the universal primers: 338 F(5′-ACTCCTACGGGAGGCAGCA-3′) and 806 R(5′-GGACTACHVGGGTWTCTAAT-3′). Cycling parameters were 98°C for two min, then 30 cycles of 98°C for 30s, 50°C for 30s, 72°C for 60s and a final extension at 72°C for 5 min. Multiplex 16S rDNA amplicon sequencing was achieved by Illumina HiSeq 2500 Sequencing platform. Original tag data were generated from the paired-end reads data. The high-quality tag data were obtained after filtering and compared with sequences in the SILVA database (http://www.arb-silva.de).

### Absolute quantitative real-time PCR (absolute qPCR)

To determine what were *S. mutans* level closely associated dental plaque bacteria in CF and SECC groups, correlation analysis was performed based on the species-level operational taxonomic units (OTUs) reflecting *S. mutans* and other species levels. Once the S. mutans closely associated bacteria were determined, the species-specific PCR primers were designed and specific DNAs of each candidate species were synthesized in a series connection manner, cloned into a PGEM-T-easy vector. After DNA sequencing, the correct clone was generated as a standard template for absolute quantitative real-time PCR determines the candidate bacteria species in clinical dental plaque samples from CF, SECC with low level of *S. mutans* and SECC with high level of *S. mutans*.

qPCR was performed in triplicate using an SYBR Green Reagent (Abclonal) and run on a Thermo Pico qPCR machine. Primers for each species are listed in Table 1. qPCR conditions were 10 min at 95°C, followed by 40 cycles of 95°C for 15 s and 60°C for 1 min. Primer pairs specific to candidate bacteria were designed and validated by sequencing and alignment of each of PCR products. The sequences of each primer pairs are listed in Table 1. Candidate species levels were calculated using 2^–ΔΔCt^ method. Standard curves of primers were obtained by measuring eight 10-fold series diluted DNA standards (targeted DNA fragment cloned in plasmid PGEM-T-easy). Reaction specificities were confirmed via melting curve analysis with a progressive increase in temperature and continuous fluorescence acquisition. The standard DNA amplification curve and melting-point product curve for each primer combination were obtained to calculate the quantity of DNA.

**Table 1.** The sequences of qPCR primer used in this study for bacteria detection

Bacteria	Forward primer	Reverse primer
*S.mutans*	CGGCAATGGACGAAAGTCTG	GTTAGCCGTCCCTTTCTGGT
*Selenomonas_3*	TCTGTTGAAGGGGACGAACG	CCAATGATTCCGGACAACGC
*Leptotrichia*	TATCGGAGAGGTGGACGGAA	TCGCACTTCAGCGTCAGTTA
*Prevotella_7*	GTAGGCCGCAGGTTAAGTGT	TTTCACCGCTACACGACGAA

### Data processing and statistical analysis

The bioinformatics analysis was conducted using QIIME. The alpha diversity indices of Chao1, ACE and Shannon and Simpson were calculated using Mothur software (version v.1.30) with coverage over 99%. Beta diversity analysis was performed by nonmetric multi-dimensional scaling and unweighted pair-group method with arithmetic mean based on the unweighted UniFrac distances. The Wilcoxon rank-sum test was used to compare the relative abundance of the bacterial species. Linear discriminant analysis (LDA) effect size (LEfSe) was conducted to define the biomarkers of the three groups. The threshold on the logarithmic LDA score for the distinguishing features was set to 4.0. We performed co-occurrence analysis through Spearman correlations for compositional data calculation according to the abundance and variation of each taxon in each sample using SPSS software. Microbial functions were predicted using PICRUSt (v1.0.0) software following the online protocol and aligned to the Clusters of Orthologous Groups of proteins (COG) database. qPCR data were analyzed with ANOVA. Level of statistical significance (*p* value) was set as <0.05.

## Results

The mean age of caries-free (CF) group, in which children who had no caries lesions or restorations, was 3.68 ± 0.36 years; and SECC group, in which children diagnosed with severe early childhood caries, was 4.08 ± 0.68 years. There was no difference between two groups in terms of gender and age based on Chi-squared analysis and the Kruskal-Wallis test. The mean value of decayed, missing due to caries, or filled teeth was seen in supplementary Table S1.

### Sequencing of oral samples

A total of 1,412,560 pairs of reads were sequenced from 20 samples, and a total of 1,106,070 reads were generated after double-ended reads quality control and splicing clean reads. At least 27,875 clean reads were produced per sample, with an average of 55,304 clean reads. The species-level operational taxonomic units (OTUs) at 3% dissimilarity for each sample are shown in supplementary Table S2. Altogether, 10 phyla, 16 classes, 30 orders, 50 families, 89 genera and 101 bacterial species were represented by all the samples.

### Bacterial community composition in CF and SECC groups

No significant difference was observed between the CF and SECC groups in community richness according to alpha diversity indices Chao1 and Ace, but the alpha diversity was significantly higher in SECC groups than CF groups when comparing Simpson and Shannon indices (Figure S1A). NMDS based on the compositions of OTUs in each sample was performed to evaluate beta diversity by comparing the overall bacterial community between the two groups. The modeling separated control from the SECC (Figure S1B). Also, a phylogenic tree drawn on the basis of the weighted Unifrac distances showed distinct patterns in CF and SECC groups (Figure S1C).

We compared the species in all samples and identified top 20 differential bacteria with the smallest p values (Figure S1D). At species level, we found most differential bacteria were uncultured, including *Tannerella, Stomatobaculum, Selenomonas_3, Selenomonas, Prevotella_7, Prevotella_6, Prevotella, Leptotrichia, F0058, Alloprevotella, Aggregatibacter and SR1_bacterium_MGEHA*, and only small amounts of species were cultured, such as *Selenomonas_sp._oral_clone_EY047 and Leptotrichia_sp._oral_clone_GT020*. All above bacteria presented a significantly higher abundance in SECC group compared to CF group. Moreover, uncultured bacteria including *Pseudopropionibacterium, Neisseria, Lautropia, Bergeyella, Abiotrophia* and cultured bacteria *Lautropia_sp._TeTO* had inverse tendencies, which presented a significantly higher abundance in CF group compared to SECC group.

### Predicted function of bacteria in CF and SECC groups

**Figure 1.**
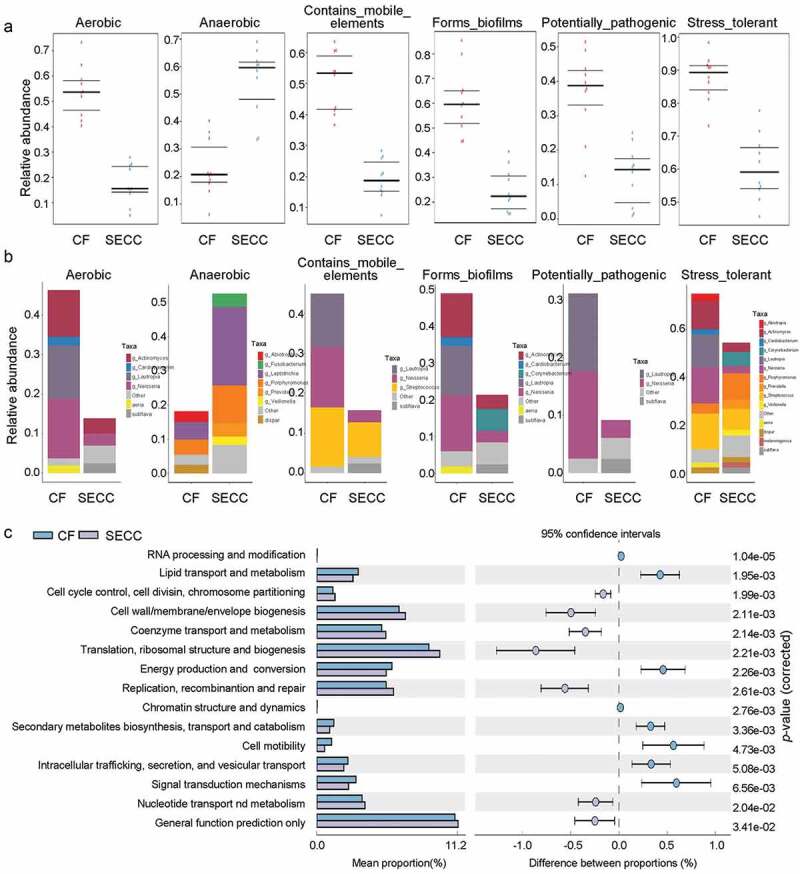
Phenotype and functional analysis of dental plaque microorganism in CF and SECC groups. A. BugBase analysis. B. Predicted phenotypes. C. COG analysis of differential bacteria between CF and SECC.

It was observed that SECC group was predicted to have the decreases in aerobic respiration, mobile elements, biofilms forming, potential pathogenicity and stress tolerance, but only an increase in anaerobic respiration (Figure 1a,b). Meanwhile, based on the abundance of bacteria, we found that *Lautropia* and *Neisseria* contributed to the phenotypes of caries free subjects. *Corynebacterium* presented a specific difference on biofilms forming and stress tolerance in SECC patients. *Fusobacterium, Leptotrichia, Porphyromonas, Prevotella and Veillonella* had higher survival ability in anaerobic environment in SECC. *Porphyromonas, Prevotella, Veillonella* and *melaninogenica* were at higher abundance on stress tolerance in SECC group (Figure 1b).

Though COG analysis of the predominant protein functional features between CF and SECC, we found that the SECC group showed significantly higher proportions in functional features, including cell cycle control, cell wall/ membrance/ envelope biogenesis, coenzyme transport and metabolism, translation, replication and repair, nucleotide transport and metabolism, general function prediction only. Moreover, it exhibited lower level of RNA processing, lipid transport, energy production, chromatin structure and dynamics, secondary metabolites biosynthesis, cell motility, intracellular trafficking and signal transport (Figure 1c).

### Bacterial community composition based on S. mutans and caries status

**Figure 2.**
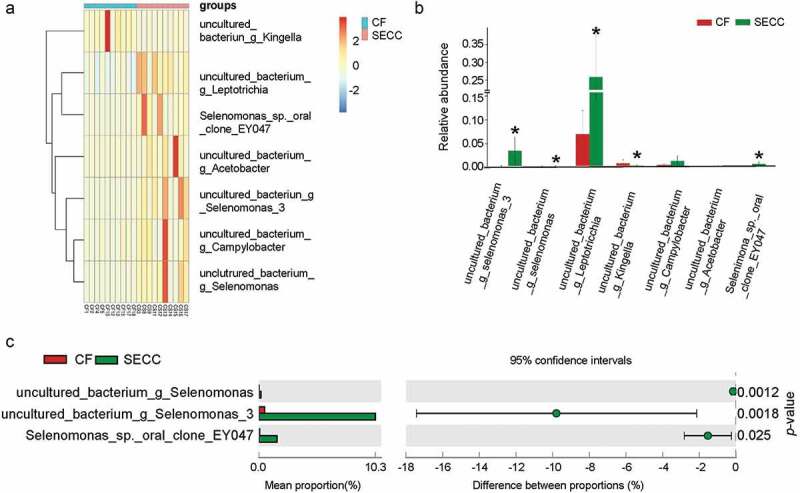
Analysis of the bacteria correlated with *S. mutans* in CF and SECC groups. According to the relative abundance of bacteria (the correlated *S.mutans* bacteria screening threshold is |R|> 0.6 and *p* < 0.05.) at species level, CF and SECC groups were analyzed for correlation with *S. mutans*. The union section of CF and SECC groups was used to determine the differential bacteria by Wilcox rank test. The differential species was utilized to draw heatmap (A) and species abundance histogram (B) based on the differential bacterial abundance of the selected species in CF and SECC groups. In addition, STAMP analysis was performed by using the union section of the two groups at species level (C).

The bacteria correlated with *S. mutans* were compared between CF and SECC (Table 2). Of all estimated bacterial taxa at the species level, seven species were detected in all samples with the largest R value. A heatmap was prepared for comparing the relative abundance of each taxon between the two groups. The result revealed markedly differential bacteria included some uncultured bacteria (*Leptotrichia, Acetobacter, Selenomonas_3, Campylobacter, Selenomonas*) and *Selenomonas_sp._oral_clone_EY047, which* were more frequently detected in SECC; *uncultured_bacterium_g_Kingella*, which was more commonly detected in CF (Figure 2a). Meanwhile, significant differences were found in *uncultured_bacterium_g_Leptotrichia, Selenomonas_sp._oral_clone_EY047, uncultured_bacterium_g_Selenomonas_3, uncultured_bacterium_g_Selenomonas* and *uncultured_bacterium_g_Kingella* between the two groups (Figure 2b). Among them, three species (*uncultured_bacterium_g_Selenomonas, uncultured_bacterium_g_Selenomonas_3* and *Selenomonas_sp._oral_clone_EY047*) were found to be enriched in SECC compared to CF (Figure 2c).

**Table 2.** The bacteria correlated with *S. mutans* in CF and SECC groups

CF				SECC			
Species	R_value	*p*_value	CF v.s. SECC (*p*_value)	Species	R_value	*p*_value	CF v.s. SECC (*p*_value)
Actinomyces_sp._oral_taxon_448_str._F0400	0.86	0.001	-	uncultured_bacterium_g_Kingella	0.89	0.001	0.008
Selenomonas_artemidis	0.85	0.002	-	uncultured_bacterium_g_Acetobacter	0.74	0.015	0.014
uncultured_bacterium_g_Selenomonas_3	0.81	0.004	0.0002	uncultured_bacterium_g_Leptotrichia	−0.68	0.030	0.001
Campylobacter_sp._oral_clone_OH5A	0.75	0.013	-	uncultured_bacterium_g_Campylobacter	−0.64	0.044	0.019
Selenomonas_sp._oral_clone_EY047	0.75	0.013	0.0003	uncultured_bacterium_g_Rothia	0.64	0.004	-
uncultured_bacterium_g_Roseburia	0.65	0.043	-	uncultured_bacterium_g_F0332	0.62	0.056	-
uncultured_bacterium_g_Prevotellaceae_UCG-003	0.64	0.045	-				
uncultured_bacterium_g_Selenomonas	0.64	0.045	0.0032				
uncultured_bacterium_g_Corynebacterium	0.62	0.056	0.0126				

Note: The union section of the bacteria correlated with *S. mutans* in CF and SECC groups was analyzed using R-psych package for Spearman correlation calculation, and the relevant bacteria screening threshold is |R|> 0.6 and *p* < 0.05. The numbers in CF vs. SECC (*p*_value) column mean the significant difference of the bacterial species between CF and SECC.

**Figure 3.**
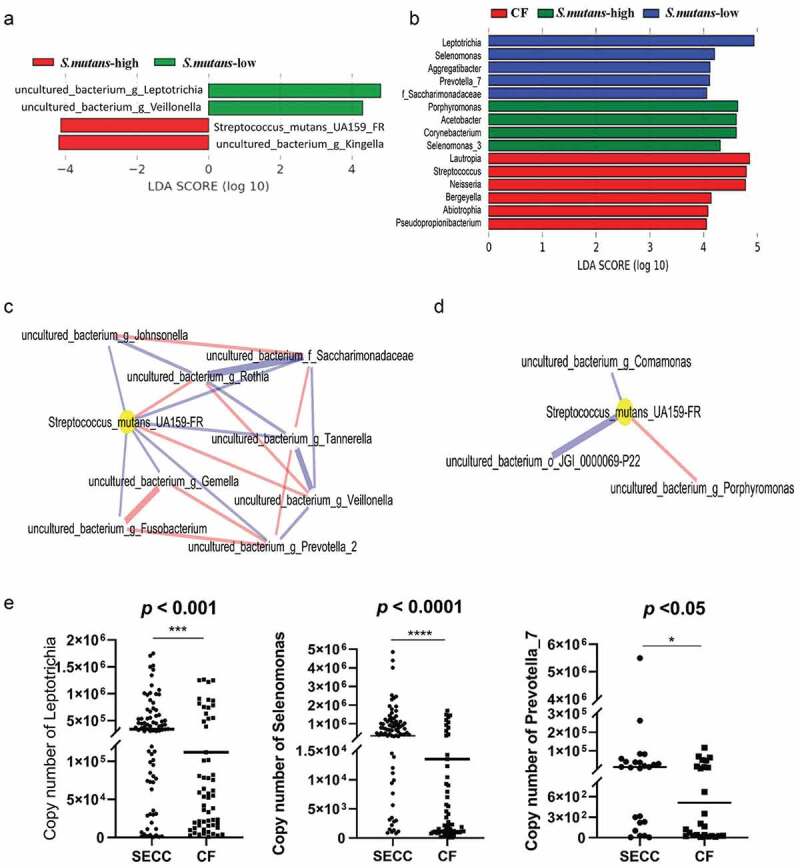
Histogram of the LDA scores for differently abundant features between groups: SECC-*S. mutans* Low and SECC-*S. mutans* High groups (A); CF, SECC-*S. mutans* Low and SECC-*S. mutans* High groups. (B). The length represents the impact. The threshold on the logarithmic LDA score for discriminative features was set to 4.0. Co-occurrence network analysis showing the interactions between predominant species (relative abundance >1%) (*p* < 0.05). Bacterial interaction of *S. mutans*-low (C); bacterial interaction of *S. mutans*-high (D). The thickness of lines represents the strength of correlations and the color of lines represents positive (red) and negative (blue) correlations. (E) The absolute abundance of target species in plaque between SECC and CF groups as measured by absolute qPCR. Mann-Whitney’s U test was used for statistical analysis. *p* < 0.05 was considered statistically significant. Asterisks denoted statistical significance (*p < 0.05;***p < 0.001; ****p < 0.0001).

At the species level, a high abundance of *S. mutans* was associated with *uncultured_bacterium_g_Kingella* in SECC group, and undetectable or extremely low levels of *S. mutans* were related to *uncultured_bacterium_g_Leptotrichia* and *uncultured_bacterium_g_Veillonella* (Figure 3a). At the genus level, *Porphyromonas, Acetobacter, Corynebacterium* and *Selenomonas_3* were significantly enriched in the SECC samples with a high level of *S. mutans*, while *Leptotrichia, Selenomonas, Aggregatibacter* and *Prevotella_7* were shown to be potential biomarkers of the SECC samples whose *S. mutans* were found either at low levels or not present. *Lautropia, Streptococcus, Neisseria, Bergeyella* and *Abiotrophia* were elected to be associated with the caries-free status (LDA > 4.0, *p* < 0.05 [Figure 3b]).

The co-occurrence network analysis using sparse correlations suggested interactions among *S. mutans* and some predominant members in the microbiota of SECC- *S. mutans* low and SECC- *S. mutans* high groups. In the SECC- *S. mutans* low group, *Streptococcus_mutans_UA159FR* had positive links to *uncultured_bacterium_g_Rothia, uncultured_bacterium_g_Veillonella* and *uncultured_bacterium_g_Prevolella*. Negative associations were observed in these bacteria including *uncultured_bacterium_g_Fusobacterium, uncultured_bacterium_g_Gemella, uncultured_bacterium_g_Tannerella, uncultured_bacterium_f_Saccharimonadaceae* and *uncultured_bacterium_g_Johnsonella* (Figure 3c). The interactions in the SECC- *S. mutans* high group were shown in Figure 3d. However, it displayed simpler relationships relative to the SECC-*S. mutans* low group. *Uncultured_bacterium_g_Comamonas* mainly present negative correlation with *Streptococcus*_*mutans_UA159FR*, and *uncultured_bacterium_g_Porphyromonas* was positively related with it.

*Verification of high-throughput data by absolute qPCR to reveal bacteria that may highly contribute to caries occurrence*

To confirm the correctness of the conclusion generated from 16S rRNA high throughput sequencing, we recruited more cases of CF and SECC to establish the validation groups. Meanwhile, we designed specific primers for each candidate species as well as universal primers for all bacteria detection. After verification of the specificity of each primer through melting curve examination via absolute qPCR detection system, PCR products were sequenced and the sequence alignment was performed via BLAST at the website (https://blast.ncbi.nlm.nih.gov/). The results showed that we successfully designed absolute qPCR primers with specificity of each bacterial species (supplementary Figure S2). Through qPCR screening, we found that, among these bacteria, *Leptotrichia, Selenomonas* and *Prevotella* had been proved their abundance were at significantly higher levels in children with SECC than that in CF group (Figure 3e), which supported our high-throughput data.

## Discussion

For decades, *S. mutans* has been considered the main causative agent of dental caries. However, recent studies uncovered that *S. mutans* was detectable with high abundance only in a part of caries cases. In some cases with caries, *S. mutans* level was low abundance or even undetectable. This means there have other bacteria must be involved in the process of caries occurrence. It has been proved that other oral species can act synergistically to increase their pathogenic effect, and some other microbial species may play a vital role in the process of tooth decay. Therefore, determining the importance of *S. mutans* in caries development requires a comprehensive consideration of its virulence factors. The abundance of species might not be the sole predictor for caries and links among species can be exploited to discriminate caries status in the models [15].

The present study characterized the microbiota in the two groups of children aged from two to six years, with a focus on profiles in caries-diseased subjects with or without *S. mutans*. Through gene sequencing approach, 16S rRNA profiling presented more anaerobic bacteria species including *Fusobacterium, Leptotrichia, Prevolella, Veillonella* contributed to the functional phenotype in SECC than CF, which were in line with previous studies [9,16–18].

Interestingly, to further analyze the data, we, for the first time, report that in subjects with caries experience, high levels of *S. mutans* were associated with the enumeration of a few bacteria species and low levels or no *S. mutans*, with more species. We proposed the explanation that *S. mutans* functioned as the dominated cariogenic bacterium through its strong capacities of acid production, acid tolerance and exopolysaccharide production, which perhaps lead to outcompeting commensal bacteria directly in SECC with high levels of *S. mutans* group, breaking the balanced links with other oral bacteria. The enrichment of dental plaque with cariogenic microorganisms is generally accompanied by the loss of less acid-tolerant, less cariogenic organisms, which are abundant in so-called healthy dental plaque [19].

Meanwhile, most of the associations between *S. mutans* and other bacteria existed in a part number of SECC patients with low abundance of *S. mutans*, just like those in CF, which indicated other bacterial species must be involved in the process of caries occurrence and need to be elucidated. Our suspection is supported by Loesche’s study [20].

In this study, we focused on *Leptotrichia, Selenomonas, Prevotella, Veillonella* and *Kingella* based on our analysis of the bacteria correlated with *S. mutans* in CF and SECC groups and differently abundant features between groups *S. mutans*-low and *S. mutans*-high groups in SECC. *Leptotrichia, Selenomonas* and *Prevotella* were significantly predominant in the SECC patients with just a low abundance of *S. mutans.*

*Leptotrichia* species are nonmotile facultative, Gram-negative fusiform bacteria. In agreement with the previous report [17], *Leptotrichia* was observed a higher relative abundance in caries patients (Figure 1e, 4e). To confirm whether it has close relationships with *S. mutans* and caries, we collected more samples from SECC children and healthy children and then extracted DNA to test the abundance of *Leptotrichia* by using the method of absolute quantitative real-time PCR. Our result clearly showed that *Leptotrichia* was negatively related with the abundance of *S. mutans* and rich in caries group. According to some previous stuies, *Leptotrichia* species colonize in oral cavity and vaginal flora, with protruding structures on the cell surface that may promote attachment [21]. The elevated colonization of these bacteria might be related to the immune dysfunction due to their virulence and thus may cause some diseases [22], including oral infections such as periodontitis [23] and autoimmune oral diseases such as oral lichen planus [24]. *Leptotrichia* was also further found it was significantly correlated with IL-1β, IL-6, IL-8 and IL-10 mRNA syntheses of oral epithelium cells [25]. Moreover, *Leptotrichia* metabolizes the isomers of sucrose and produce acid, which means *Leptotrichia* perhaps has highly saccharolytic and cariogenic potential [26,27]. Therefore, we deduced that *Leptotrichia* species could be used for caries risk prediction in SECC children with a low level or no *S. mutans*.

*Selenomonas* was predicted as another caries risk biomaker of SECC group with low level or without *S. mutans* in our study. Consistent with our results, a previous report showed that levels of *Selenomonas spp*. were found at relatively high levels in some subjects whose samples did not contain *Lactobacillus* or *S. mutans* [28]. *Selenomonas* may play a potentially complex role in caries progression. In previous studies, uncultivated *Selenomonas* species were associated with root caries in elderly patients [29] and coronal caries in young children [30]. The oral species *S. sputigena* has been shown to grow on lactate and to produce acetate, propionate and succinate [31]. *Selenomonas* species has been shown to both ferment glucose and utilize lactate in studies of rumen bacteria [32].

*Prevotella* is anaerobic Gram-negative bacteria. Our study showed that *S. mutans* presented a negative relationship with *Prevotella* when *S. mutans* was detected at low levels and even nonexistent, whereas this link disappeared when *S. mutans* was at high levels in individuals with SECC. A previous study showed the same tendency [15]. Besides, previous studies have unveiled *Prevotella* spp*’*s close relationship with caries [33,34]. Being able to overexpress collagenases for proteolytic metabolism in *Prevotella* species may lead to the progression of dental caries with accuracy of 74% [18]. In addition, a caries risk assessment model based on the relative abundance of seven *Prevotella spp*. has been used to predict new onsets of ECC [7]. The result reminds us to pay more attention to some other bacterial members which may replace *S. mutans’*s leading role in acid-producing and acid-resisting to cause SECC.

*Veillonella*, which belongs to Gram-negative obligate anaerobic coccus, cannot metabolize carbohydrates and polyols, but use short-chain organic acids, especially lactic acid as energy, to transform to less acidic acetic acid and propionic acid. This may imply a beneficial effect. But in some previous reports, *Veillonella* is proved a close association with caries. *Veillonella* is detected to be significantly related with caries in children [9,35,36]. In our study, we also found that *Veillonella* showed significant difference between SECC-*S. mutans*-low and SECC-*S. mutans*-high groups. It has been shown that combinations of *S. mutans* and *Veillonella* could have higher incidence of caries by promoting the growth and exopolysaccharide synthesis of *S. mutans* [37]. It means that *Veillonella* may help to promote the toxicity of *S. mutans* in the process of caries development though *S. mutans* is not a dominant species in such individuals of caries. Thus, the contribution of *Veillonella* to caries remains incompletely conclusive, but the current studies prefer to recognize the important role of it in the caries-causing process.

Uncultured bacterium of *Kingella* is assigned as *Kingella oralis* by NCBI. Although Cherkasov et al. shows that *Kingella oralis* is one of the statistically significant caries-enriched species [38], other studies deduce it is included in the healthy microbiota [39,40].

In contrast to some findings of previous reports that *Corynebacterium matruchotii* and *Corynebacterium durum* were abundant in the naturally healthy adult and children [17,41], our result showed that *Corynebacterium* was enriched in SECC-*S. mutans* high group. This result was similar to the recently published reference [42]. Based on the relationship between its abundance and phenotypes, we deduced that *Corynebacterium* exhibited contribution to biofilms forming and stress tolerance in the process of caries occurrence.

Besides the above bacteria, we also found that *S. mutans* was associated with many uncultured bacterial species. *S. mutans* exhibited a high degree of negative linkages with *uncultured_bacterium_g_Fusobacterium, uncultured_bacterium_g_Gemella, uncultured_bacterium_g_Tannerella, uncultured_bacterium_f_Saccharimonadaceae* and *uncultured*_*bacterium_g_Johnsonella*, most of which are poorly characterized species. The results indicate the complexity of dental plaque microorganism, cariogenic mechanism remains largely unknown. Although the limited samples were used in this study, we still found many cultivated or noncultivated bacteria associated with the level of *S. mutans*, which may contribute to a novel strategy for caries prevention. Of course, expansion of the sample size and deep investigation of the potential etiologic roles of the *S. mutans*-associated bacteria and the diverse bacterial communities are needed in future.

In summary, we, for the first time, found the potential cariogenic bacteria including *Leptotrichia, Selenomonas and Prevotella* in the SECC patients with just a low abundance of *S. mutans*. They are identified as candidate biomarker in SECC with low abundance or without *S. mutans*. Our study may shed light on the understanding of caries occurrence in SECC with low *S. mutans*.

## Supplementary Material

Supplemental Material
