# Supplementary material for: Streptococcus mutans-associated bacteria in dental plaque of severe early childhood caries
Source: J Oral Microbiol. 2022 Mar 2;14(1):2046309. doi: 10.1080/20002297.2022.2046309 (PMC8896182; doi:10.1080/20002297.2022.2046309)
Supplement: Supplemental Material [file ZJOM_A_2046309_SM6497.zip › Supplementray/Supplementary Legends.docx]

**Supplemental Information**

**Table S1**. Characteristics of samples in the 2 Study Groups.

Note: ^*^dmft (d: decayed, m: missing, or f: filled t: deciduous teeth).

**Table S2**. The species-level operational taxonomic units (OTUs) for samples in CF and SECC groups

**Figure S1** A.Alpha diversity. ** *p* < 0.001. B.beta diversity. C.weighted unifrac NMDS and weighted unifrac upgram tree. D. The relative abundance of differential bacteria between CF and SECC groups. Bars indicate standard error. **p* < 0.05, Wilcoxon rank-sum test.

**Figure S2** Blast results showing the qPCR products are matched with the specificity of *Leptotrichia*(A), *Selenomonas_3*(B), *Streptococcus mutans*(C), *Prevotella_7*(D).
