# Supplementary material for: Streptococcus mutans-associated bacteria in dental plaque of severe early childhood caries
Source: J Oral Microbiol. 2022 Mar 2;14(1):2046309. doi: 10.1080/20002297.2022.2046309 (PMC8896182; doi:10.1080/20002297.2022.2046309)
Supplement: Supplemental Material [file ZJOM_A_2046309_SM6497.zip › Supplementray/Table S1.docx]

Table S1. Characteristics of samples in the 2 Study Groups.

| Sample ID | Gender | Age(year) | dmft^*^ | |
| --- | --- | --- | --- | --- |
| CF1 | Female | 3.45 | 0 |  |
| CF2 | Female | 3.36 | 0 |  |
| CF4 | Male | 3.46 | 0 |  |
| CF5 | Male | 4.51 | 0 |  |
| CF10 | Male | 3.66 | 0 |  |
| CF12 | Male | 3.93 | 0 |  |
| CF13 | Female | 3.27 | 0 |  |
| CF15 | Female | 3.69 | 0 |  |
| CF17 | Male | 3.89 | 0 |  |
| CF18 | Female | 3.59 | 0 |  |
| SECC5 | Male | 5.09 | 16 |  |
| SECC8 | Female | 4.67 | 14 |  |
| SECC9 | Male | 3.42 | 18 |  |
| SECC11 | Male | 3.74 | 12 |  |
| SECC12 | Male | 4.05 | 13 |  |
| SECC13 | Female | 3.25 | 12 |  |
| SECC14 | Female | 4.96 | 13 |  |
| SECC15 | Male | 3.98 | 14 |  |
| SECC16 | Male | 4.40 | 16 |  |
| SECC17 | Female | 3.24 | 15 |  |

Note: ^*^dmft (d: decayed, m: missing, or f: filled t: deciduous teeth).
