# Supplementary material for: Streptococcus mutans-associated bacteria in dental plaque of severe early childhood caries
Source: J Oral Microbiol. 2022 Mar 2;14(1):2046309. doi: 10.1080/20002297.2022.2046309 (PMC8896182; doi:10.1080/20002297.2022.2046309)
Supplement: Supplemental Material [file ZJOM_A_2046309_SM6497.zip › Supplementray/Table S2.docx]

Table S2. The species-level operational taxonomic units (OTUs) for samples in CF and SECC groups

| Sample | Kindom | Phylum | Class | Order | Family | Genus | Species |
| --- | --- | --- | --- | --- | --- | --- | --- |
| CF1 | 1 | 8 | 11 | 18 | 32 | 45 | 56 |
| CF2 | 1 | 7 | 10 | 20 | 33 | 43 | 48 |
| CF4 | 1 | 7 | 10 | 20 | 33 | 48 | 52 |
| CF5 | 1 | 9 | 13 | 24 | 38 | 56 | 67 |
| CF10 | 1 | 8 | 11 | 20 | 31 | 46 | 50 |
| CF12 | 1 | 7 | 11 | 19 | 30 | 47 | 56 |
| CF13 | 1 | 7 | 12 | 21 | 34 | 47 | 52 |
| CF15 | 1 | 9 | 13 | 21 | 34 | 51 | 59 |
| CF17 | 1 | 7 | 10 | 18 | 28 | 41 | 53 |
| CF18 | 1 | 7 | 10 | 17 | 28 | 37 | 42 |
| SECC5 | 1 | 8 | 12 | 21 | 34 | 53 | 61 |
| SECC8 | 1 | 8 | 13 | 20 | 32 | 46 | 54 |
| SECC9 | 1 | 8 | 13 | 22 | 35 | 55 | 65 |
| SECC11 | 1 | 7 | 12 | 20 | 33 | 54 | 65 |
| SECC12 | 1 | 8 | 12 | 22 | 35 | 52 | 63 |
| SECC13 | 1 | 8 | 13 | 24 | 36 | 58 | 67 |
| SECC14 | 1 | 8 | 11 | 20 | 31 | 47 | 57 |
| SECC15 | 1 | 8 | 13 | 21 | 33 | 53 | 61 |
| SECC16 | 1 | 8 | 13 | 21 | 33 | 50 | 60 |
| SECC17 | 1 | 8 | 12 | 21 | 32 | 51 | 61 |
| Total | 1 | 10 | 16 | 30 | 50 | 89 | 101 |
