# Supplementary figures and images for: Streptococcus mutans-associated bacteria in dental plaque of severe early childhood caries
Source: J Oral Microbiol. 2022 Mar 2;14(1):2046309. doi: 10.1080/20002297.2022.2046309 (PMC8896182; doi:10.1080/20002297.2022.2046309)

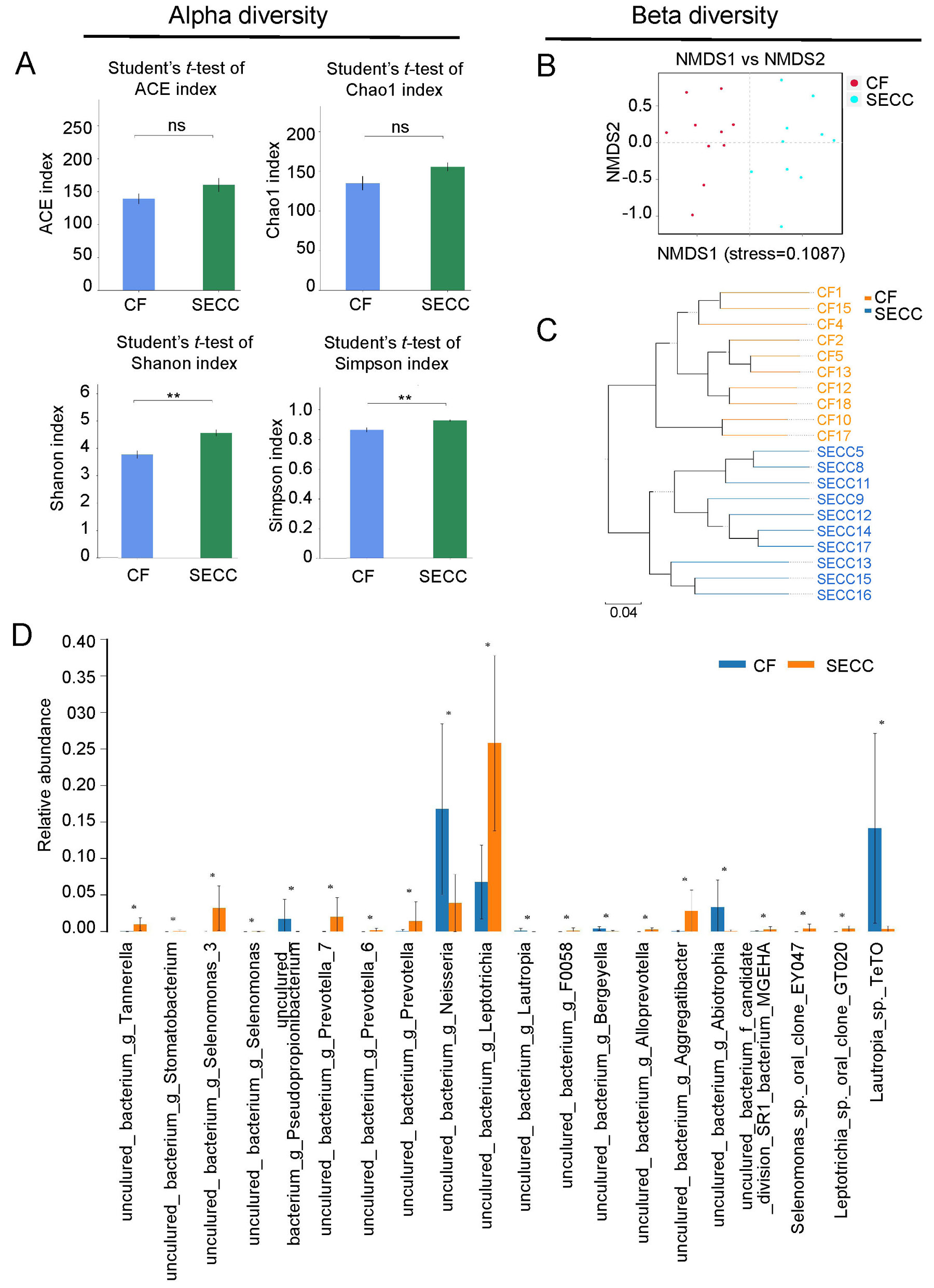

Supplement: Supplemental Material [file ZJOM_A_2046309_SM6497.zip › Supplementray/Figure S1 final.jpg]

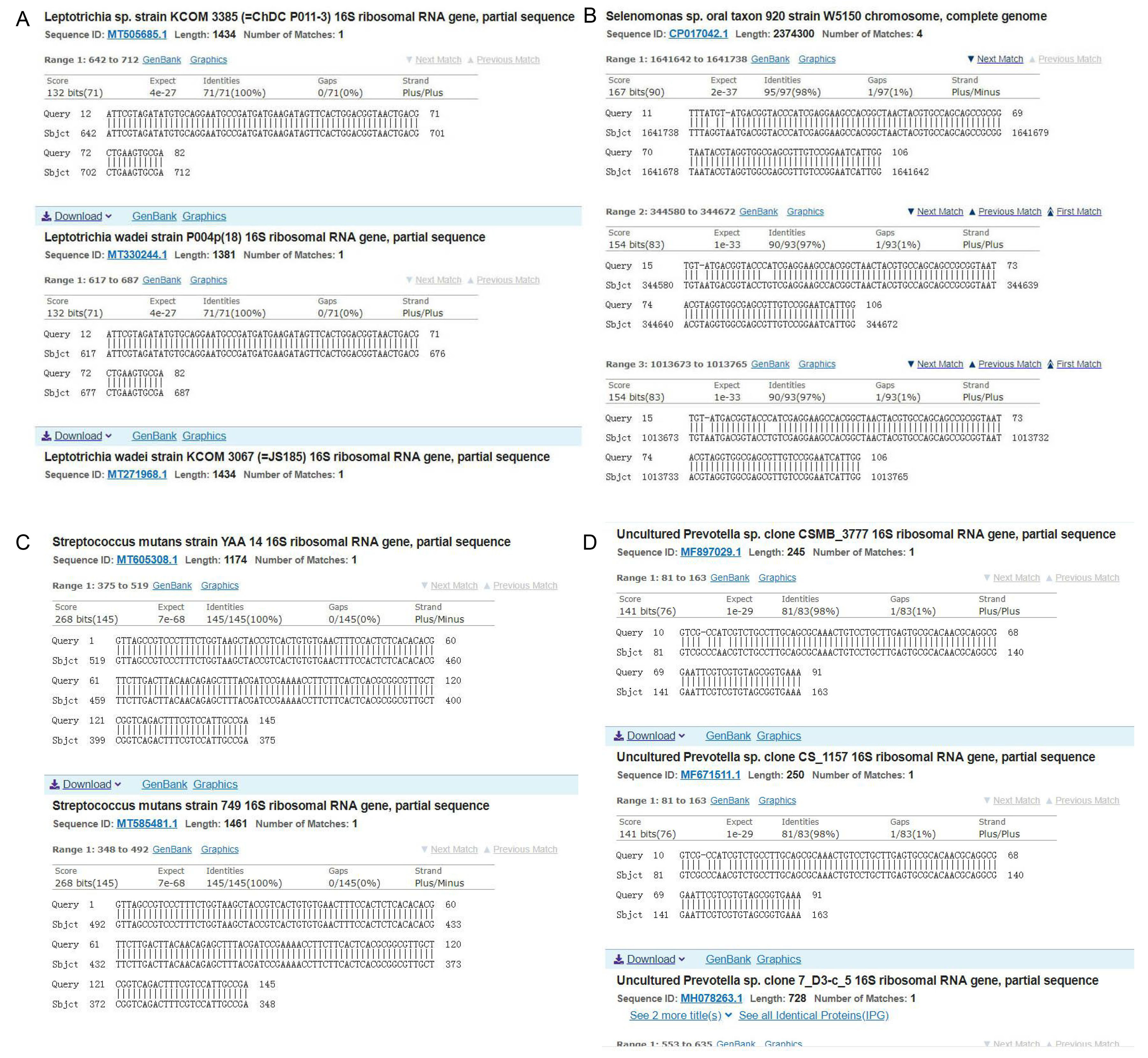

Supplement: Supplemental Material [file ZJOM_A_2046309_SM6497.zip › Supplementray/FigureS2.jpg]
